# Imported malaria cases: the connection with the European ex-colonies

**DOI:** 10.1186/s12936-019-3042-1

**Published:** 2019-12-04

**Authors:** Marta Domínguez García, Cristina Feja Solana, Alberto Vergara Ugarriza, Cruz Bartolomé Moreno, Elena Melús Palazón, Rosa Magallón Botaya

**Affiliations:** 1 Aragonese Primary Care Research Group, IIS (Instituto de Investigación Sanitaria Aragón) GIIS-011, 50015 Zaragoza, Spain; 2 Aragonese Health Service, Zaragoza, Spain; 3 General Directorate of Public Health, Aragon Government, Zaragoza, Spain; 4 Aragonese Primary Care Research Group, B21-17R, Aragon Government, Zaragoza, Spain; 5 University of Zaragoza, Zaragoza, Spain

**Keywords:** Malaria, Epidemiology, Immigrants, Sanitary control of travelers, Spain, Public health

## Abstract

**Background:**

Imported malaria is increasing in non-endemic areas due to the increment of international travels, migration and, probably, other unknown factors. The objective of this study was to describe the epidemiological and clinical characteristics of malaria cases in a region of Spain; analyse the possible association between the variables of interest; compare this series with others; and evaluate the characteristics of imported malaria cases according to the country of origin, particularly cases from Equatorial Guinea (Spanish ex-colony) and from the rest of sub-Saharan Africa.

**Methods:**

A descriptive observational study was carried out with a retrospective data collection of cases of malaria reported in Aragon from 1996 to 2017. Univariate and bivariate analysis of clinical–epidemiological variables was performed. In addition, an analysis of cases from sub-Saharan Africa was carried out using logistic regression, calculating odds ratio with its 95% confidence interval.

**Results:**

609 cases of malaria were recorded in Aragon from 1996 to 2017. An autochthonous case in 2010. 50.33% were between 15 and 39 years old. 45.65% of the cases were notified of the 4-weeks 9 to 12. 82.6% reside in the main province, urban area, of which 65.4% were VFR (Visiting Friends and Relatives), 23.8% new immigrants and 10.9% travellers. The infectious *Plasmodium* species par excellence was *Plasmodium falciparum* (88%). Analysing the cases from sub-Saharan Africa (95.2% of the total), 48.1% were from Equatorial Guinea. Comparing these with the cases from the rest of sub-Saharan Africa, it was observed that the cases from the Spanish ex-colony have association with the female gender, being under 5 years old, residing in the main province (urban area) and being a new immigrant.

**Conclusions:**

The epidemiological profile of imported malaria cases can be defined as VFR between 15 and 39 years old, coming from sub-Saharan Africa, particularly from Equatorial Guinea. Immigrants education about the importance of chemoprophylaxis when travelling to visit friends and relatives, emphasizing on those who are originally from the ex-colonies of destination country, is necessary; as well as to raise awareness among health professionals to make advice in consultations, specially before summer vacations.

## Background

According to the World Health Organization [1], in 2016, 91 countries reported a total of 216 million cases of malaria, an increase of 5 million cases in relation to the previous year. The total number of deaths worldwide reached 445,000, similar to that reported in 2015. Although the incidence of malaria cases has decreased worldwide since 2010, the rate of decline has stagnated and even reversed in some regions since 2014. During the last century, more than 50 countries managed to eradicate the disease; however, although malaria has ceased to be an endemic disease in these countries (developed countries), the increase in trips to endemic areas in recent decades means that imported malaria cases are becoming more common [1–3], as is the case in Spain where every year there are more than 500 cases of imported malaria [4]. On the other hand, vector-borne transmission in non-endemic areas is unlikely, but possible because of the establishment of *Anopheles* species in new areas [5]. For example, in October 2010, an autochthonous case of malaria was registered in Spain, of a 48-year-old woman who fell ill due to *Plasmodium vivax* infection [6].

It has been shown that sub-Saharan immigrants who return to see their friends and relatives (VFR) are more likely to be affected, most of them without taking correct chemoprophylaxis [7–9]. Although malaria can be prevented and treated, it continues to have devastating effects on the health and lifestyle of people around the world.

Previous cohort studies have established that the most common origin of the imported malaria cases in Spain is the ex-colony of Equatorial Guinea [7, 8, 10, 11]. Also in other non-endemic countries, there has been a higher prevalence of malaria imported from their corresponding ex-colonies [12–14], although the percentage of the ex-colony population resident in each non-endemic country is not greater than the percentage of population resident originated from other endemic countries that are not ex-colonies. The air traffic network (high-traffic routes), as well as historical and linguistic links have been shown to be related to cases of imported malaria [15].

Recently, a research about imported malaria cases in Spain using global nationally reported surveillance data, has been published [16], but the study period is from 2002 to 2015 (while the present study is longer and more recent), and there are differences in some variables evaluated (e.g. continents not countries, without analysis of the Equatorial Guinea cases).

The general objective of this study is to know the most relevant epidemiological characteristics of the malaria cases reported in a region of Spain (Aragon) during the years 1996 to 2017; the possible association between the variables of interest, and to know possible differences between the cases coming from Equatorial Guinea and those of the rest of sub-Saharan Africa trying to identify the possible causes of the higher prevalence of cases from this ex-colony.

## Methods

A descriptive observational study design was implemented with retrospective data collection, to examine all malaria cases reported to the Public Health Agency of Aragon between 1996 and 2017. Malaria is a notifiable disease in Spain. The Aragon Malaria database was accessed, provided by the Public Health Agency under numerical coding. In addition, the main researcher reviewed the epidemiology surveys reported in the main province of the region to obtain information from some variables of interest for the study. Only the surveys declared since 2000 were conserved. This main province includes 73% of the population, specially urban area (Zaragoza). There were no exclusion criteria.

The following variables were studied: year of notification, epidemiological week, gender, age, place of residence rural or urban, endemic geographical region visited, *Plasmodium* species, hospitalization, death, chemoprophylaxis, presence of fever at diagnosis and reason for travel: immigrant (people from endemic countries that have just arrived in Spain); VFR (immigrant who live in Aragon and return to the country of origin); traveller (people from non-endemic region who travel to endemic country less than 3 months, including people who travel for tourism, cooperation or work). Of the last three variables, information is available only on the cases notified in the main province from 2000 to 2017.

Univariate descriptive analysis of qualitative variables was performed by frequencies and percentages, and quantitative variable (age) by median and interquartile range (IR) according to their no normal distribution. Incidence rates were calculated per 100,000 person-years according to the total population of Aragon in each period. At the bivariate level, the chi-test was used to evaluate the association between two qualitative variables, or Fisher’s exact test in the case of dichotomous qualitative variables (2 × 2 tables) that did not meet the conditions of chi-test application. In all cases it was considered that the results reach statistical significance with p < 0.05. In addition, the strength of association was measured by the Phi and Cramérs V coefficients, as well as the Yule coefficient Q for the 2 × 2 tables; and by means of the contingency coefficient and the typified residuals for the rest of the tables. At the multivariate level, logistic regression was used, comparing immigrants from Equatorial Guinea to those from the rest of sub-Saharan Africa. Odds ratio (OR) and its 95% confidence interval (95% CI) were calculated and a significance level of 5% (p < 0.05) was accepted as statistically significant. Statistical analyses were performed using SPSS version 22.0.

## Results

**Fig. 1:**
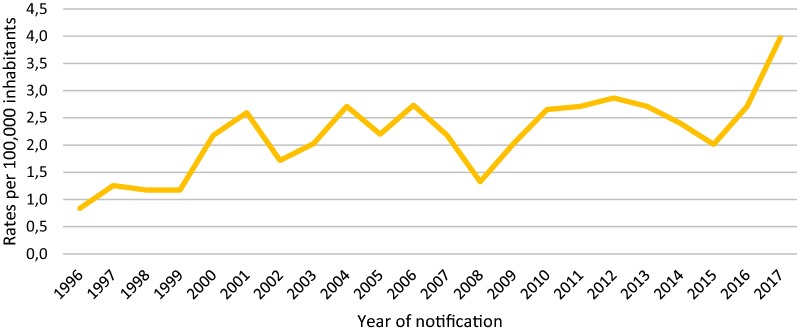
Evolution of malaria incidence rates per 100,000 inhabitants in Aragon

A total of 609 cases were notified during the 22 year study period. Figure 1, shows the evolution of malaria incidence rates per 100,000 inhabitants in Aragon. A stable trend with ups and downs is observed from 2000 to 2015, especially in 2008, the year in which the economic crisis began. The notable increase from 2015 is striking, being 2017, the last year of the series, the year with the highest number of cases (52).

The main characteristics of malaria cases are described in Table 1. There are two age groups that stand out in terms of the number of cases, 2 years (N = 28, 4.6%) and 32 years (N = 26, 4.3%), observing in the histogram a bimodal distribution. Median age of 30 years old (IR 26.25), with age grouping, 50.33% were between 15 and 39 years old. 22.44% were older than 39 years, 15.34% were between 5 and 14 years and 11.89% under 5 years old. The cases were predominantly from Africa, VFR and infected by *P. falciparum*. 45.65% of the cases were reported in the epidemiological 4-week period 9 to 12, corresponding to the months of August to November, after the summer holidays.

**Table 1:** Main characteristics of malaria cases

Cases in the region of Aragon (1996–2017) (N = 609)	
Age (median)	30 (IR 26.25)
Sex: male/female; N (%)	354 (58.1%)/255 (41.9%)
Continent of travel; N (%)	
Africa	538 (95.2%)
Latin America	13 (2.3%)
Asia	12 (2.1%)
Oceania	1 (0.2%)
Europe^a^	1 (0.2%)
*Plasmodium* species; N (%)	
*P. falciparum*	475 (88%)
*P. vivax*	28 (5.2%)
*P. ovale*	7 (1.3%)
*P. malariae*	9 (1.7%)
Mixed infection	4 (0.7%)
*Plasmodium* sp.	17 (3.1%)
Hospitalization; N (%)	
Yes	540 (89.26%)
No	65 (10.64%)
Death; N (%)	
Yes	4 (0.7%)
No	570 (99.3%)
Cases in the main province (2000–2017) (N = 459)	
Reason for travel; N (%)	
Immigrant	109 (23.8%)
VFR	300 (65.4%)
Traveller	
Tourism	22 (4.8%)
Work	19 (4.0%)
Cooperation	9 (2.0%)
Chemoprophylaxis; N (%)	
No	329 (74.9%)
Yes	73 (16.6%)
Incomplete	37 (8.4%)
Fever at diagnosis; N (%)	
Yes	388 (85.5%)
No	66 (14.5%)

The lost cases of each variable are not included in the table

*IR* interquartile range

^a^Corresponding to the only autochthonous case reported in Aragon, Spain

Table 2 presents the association between the reason for travel and other variables of interest. There is an association between being VFR and *P. falciparum* infection, as well as being an immigrant and the infection by other *Plasmodium*. Analysing the association between the *Plasmodium* species and the continent of travel, a strong association was observed (p < 0.001; Q Yule 0.89) between infection with *P. falciparum* and travel to Africa, as well as infection with other *Plasmodium* species and other continents of travel (Asia and Latin America).

**Table 2:** Main characteristics of malaria cases according to the travel reason, 2000–2017

	Immigrants N = 109	VFR N = 300	Travellers N = 50	Total N = 459	p value
Sex; N (%)					
Male	51 (46.8%)	163 (54.3%)	32 (64%)	246 (53.6%)	0.118
Female	58 (53.2%)	137 (45.7%)	18 (36%)	213 (46.4%)	
Age (years); N (%)					
< 5	18 (16.7%)	35 (11.7%)	3 (6%)	56 (12.2%)	< 0.001
5–14	32 (29.6%)^a^	40 (13.3%)	0 (0%)	72 (15.7%)	
15–39	43 (39.8%)	153 (51%)	33 (66%)^a^	229 (50%)	
≥ 40	15 (13.9%)	72 (24%)	14 (28%)	101 (22.1%)	
Continent of travel; N (%)					
Africa	100 (91.7%)	296 (98.7%)	42 (84%)	438 (95.4%)	–
Latin America	3 (2.58%)	1 (0.3%)	6 (12%)	10 (2.2%)	
Asia	6 (5.5%)	3 (1%)	2 (4%)	11 (2.4%)	
Plasmodium species; N (%)					
*P. falciparum*	88 (85.4%)	257 (93.5%)^a^	39 (84.8%)	284 (90.6%)	0.022
Other *Plasmodium*	15 (14.6%)^a^	18 (6.5%)	7 (15.2%)	40 (9.4%)	
Chemoprophylaxis; N (%)					
Yes	No proceed	40 (14%)	16 (32.7%)^a^	56 (16.8%)	0.001
No		216 (75.8%)^a^	25 (51%)	241 (72.2%)	
Incomplete		29 (10.2%)	8 (16.3%)	37 (11.1%)	
Fever at diagnosis; N (%)					
Yes	75 (69.4%)	266 (89.9%)^a^	47 (94%)	388 (85.5%)	< 0.001
No	33 (30.6%)^a^	30 (10.1%)	3 (6%)	66 (14.5%)	

The lost cases of each variable are not included in the table

^a^Variables that obtained statistically significant association

As previously seen, the majority of cases had travelled to sub-Saharan Africa (538 cases, 95.2%), of which 45.8% travelled to Equatorial Guinea. However, in the census of Aragon on January 1st, 2017 [17], the number of foreigners residing in this region from Equatorial Guinea was clearly lower than that of the rest of sub-Saharan Africa (5.78% from Equatorial Guinea, 94.22% from the rest of sub-Saharan Africa). Table 3 represents, at the bivariate level, the main characteristics of malaria cases comparing the cases from Equatorial Guinea to those from the rest of sub-Saharan Africa.

**Table 3:** Main characteristics of malaria cases of Equatorial Guinea versus the rest of sub-Saharan Africa

	Equatorial Guinea	Rest of sub-Saharan Africa	p value
Cases in the region of Aragon, Spain (1996–2017) (N = 538)			
	N = 259	N = 279	
Sex; N (%)			
Male	106 (40.9%)	211 (75.6%)^a^	< 0.001
Female	153 (59.1%)^a^	68 (24.4%)	
Age (years); N (%)			
< 5	41 (16%)^a^	21 (7.5%)	0.009
5–14	46 (17.9%)	41 (14.7%)	
15–39	115 (44.7%)	150 (53.8%)^a^	
≥ 40	55 (21.4%)	67 (24%)	
*Plasmodium* species; N (%)			
*P. falciparum*	228 (90.5%)	220 (91.3%)	0.755
Other *Plasmodium*	24 (9.5%)	21 (8.7%)	
Place of residence			
Urban	244 (94.2%)^a^	202 (72.4%)	< 0.001
Rural	15 (5.8%)	77 (27.6%)^a^	

Cases in the main province (2000–2017) (N = 438)			
	N = 238	N = 200	
Reason for travel; N (%)			
Immigrant	68 (28.6%)^a^	32 (16.0%)	0.001
VFR	155 (65.1%)	141 (70.5%)	
Traveller	15 (6.3%)	27 (13.5%)^a^	
Chemoprophylaxis; N (%)			
Yes	41 (17.7%)	29 (15.5%)	0.837
No	171 (73.7%)	141 (75.4%)	
Incomplete	20 (8.6%)	17 (9.1%)	

The lost cases of each variable are not included in the table

^a^Variables that obtained statistically significant association

At the multivariate level, it was observed that imported malaria among the Equatorial Guinea cases was associated with being women (OR 3.89, 95% CI 2.65–5.72), younger than 5 years (OR 2.11, 95% CI 1.06–4.24), being immigrant (OR 2.70, 95% CI 1.19–6.14) and residing in the main province (urban area) (OR 4.93, 95% CI 2.65–9.20).

Only 13 cases were from Latin America (2.3%), of which were: 6 men (46.2%) and 7 women (53.8%); 10 between 15 and 39 years old (76.9%) and 3 older than 40 years (23.1%); 9 cases caused by *P. falciparum* (69.2%) and 4 by *P. vivax* (30.8%); 3 inmigrants (30%), 1 VFR (10%) and 6 travellers (60%). Coming from Asia there were 12 cases (2.1%), of which were: 6 men and 6 women (50%); 3 younger than 5 years old (25%), 1 between 5 and 14 (8.3%), 7 between 15 and 39 (58.3%), and 1 older than 40 years old (8.3%); 12 (100%) were caused by *P. vivax*; 6 inmigrants (54.5%), 3 VFRs (27.3%) and 2 travellers (18.2%). All cases from Latin America and Asia had fever at diagnosis.

## Discussion

The epidemiological profile of malaria cases in this series is: young individual between 15 and 39 years old, VFR, from sub-Saharan Africa (particularly from Equatorial Guinea), and resident in the main province which is mostly urban. This patient profile coincides with that observed in other studies [7, 18–20], included that published recently [16].

The increase on the imported malaria cases in non-endemic areas like Spain has been described [1, 16], but since these reports end in 2015, they do not show the pronounced more recent increase, with a peak in 2017. The reason for this increment is unclear; it could be due to social changes that involve making more trips to tropical areas, perhaps there is less and less awareness of prevention. What is clear is that it is necessary to stop this increase.

Aragonese cases are distributed in a greater percentage (45.65%) during the 4-week period 9 to 12, corresponding to the months of August to November, months after the summer holidays in Spain. Some studies done in the northern hemisphere have observed a higher percentage of cases reported in the months of July, August and September, corresponding to summer holidays (boreal summer) [20–23]. On the other hand, Pagès et al. [24], analysing cases of imported malaria in Réunion Islands, observed a greater number of cases in the months of January and February, corresponding to summer holidays in the southern hemisphere (austral summer). Therefore, it can be said that the cases of imported malaria are registered mostly after the summer holidays, as it is the season that concentrates the greatest number of movements of the population. Accordingly, clinicians should pay greater attention and actively search for potential travellers prior to these months for advice and chemoprophylaxis.

87.96% of the cases had as causal agent *P. falciparum*. This percentage resembles what has been observed in other studies conducted in Spain, with 77.46%, 84.1% or 86.43% of imported malaria cases caused by *P. falciparum*, respectively [7, 8, 10]. The case-fatality rate of malaria cases reported in Aragon from 1996 to 2017 is 0.7%, which coincides with that observed in other studies, between 0.2 and 3% [16, 25]. However, there is no similarity in the percentage of Aragonese cases that required hospital admission, with a high percentage of income (89.3%) compared to 22.1% of Millet et al. [8]. This could be due to the fact that the cases analysed in this study were notified in Reference Units for Tropical Diseases, with better knowledge of malaria management, as well as better access to diagnostic tests and antimalarial treatments. In some Spanish cities, mainly Madrid and Barcelona, there are Reference Units of Tropical Diseases, where people go before and after their tropical travels. These units have their own laboratory, professionals specialized in tropical medicine and the necessary drugs for treatments. Not in all regions of Spain have such facilities. In Aragon, there is an international traveller service consultation for vaccination, but not a reference consultation for diseases after tropical travel. This consultation is carried out in primary care centres or hospital emergencies. The laboratory test for malaria is only available in some hospitals, where hospitalization is often just be done to complete the diagnosis, continuing later outpatient treatment.

Although *P. falciparum* is the major causative agent in cases of imported malaria in both VFRs, immigrants and travellers, the percentages are different. 93.5% of the infections in the VFR group were caused by this species, with 85.4% among immigrants and 84.8% among travellers. Observing the distribution by continent, 98.7% of VFRs came from Africa, being this percentage lower among immigrants (91.7%) and travellers (84%). This difference in contagion continent may explain the association observed between being VFR and *P. falciparum* infection, as well as being an immigrant and the infection by other *Plasmodium* species, since it is in Latin America and Asia where *Plasmodium vivax* (second most frequent species of *Plasmodium* among the cases reported in Aragon) is found mostly.

Regarding the presence of fever at diagnosis, 94% of travellers presented it, decreasing to 89.9% in VFRs and 69.4% in immigrants. It has been observed statistically significant association between being VFR and presence of fever, as well as being an immigrant and the absence of it; as in other studies [7, 10]. This could be due to immigrants presenting some degree of semi-immunity against infection, secondary to previous episodes of malaria during their stay in the country of origin. As Phillips et al. [26] observed, travellers to endemic areas of malaria who are exposed to the parasite for the first time, whether children or Europeans without specific immune memory, are more vulnerable and have a higher risk of serious or even fatal malaria. In this sense, the last two mortal cases of malaria in Aragon occurred in a Spanish man who travelled to Guinea for work (2016), and in a 14-year-old boy born in Spain, of Gambian parents, who travelled to see the family for the first time (2017).

It is striking that 74.94% of the cases did not take chemoprophylaxis. This data does not imply the absence of chemoprophylaxis in general, since it is not analysing all VFRs and travellers who have gone to endemic areas, but those who have acquired malaria on that trip. This means that 16.63% of reported cases suffered malaria despite having correctly taken chemoprophylaxis. In other studies, this percentage drops to 8.7% [10]. In addition, in this series, 8.43% took chemoprophylaxis incompletely. Another interesting reading with these data is the high percentage of travellers and VFRs who travel without taking chemoprophylaxis. Only counting the individuals who acquired the infection, we observed a total of 241 cases that did not take it; without having data of all the travellers and VFRs that did not take it, but neither acquired the infection. These data are hopeless, because malaria is a disease that with the correct taking of chemoprophylaxis would greatly decrease its incidence. Roca et al., in a study conducted in the field of primary care with the aim of analysing the knowledge of the immigrant population about the need to receive health advice before travelling to their countries of origin, observed how VFRs do not usually request it because they consider it unnecessary and, when they request it, they frequently address their family doctor in the first instance [9]. Recently, the National Travel Health Network and Centre (NaTHNaC) has seen pharmacists and pharmacies in the UK extend their scope and portfolio to include travel health; moreover, atovaquone/proguanil is available without prescription [27]. Obtaining advice on travel health issues from a pharmacist could be a better option for many, because of the easier access, with flexible appointment times. It would be interesting to adopt this measure in other countries. In Spain, chemoprophylaxis for VFRs is subsidised, with prescription by any doctor of the health system. With the exception of mefloquine, which is dispensed only in the traveller’s consultation (in which there is difficulty of access by waiting list), but free of charge..

95.2% of the cases of malaria reported in Aragon from 1996 to 2017 come from sub-Saharan Africa and, of these, almost half (48.1%) from Equatorial Guinea, Spanish ex-colony. However, the number of foreigners residing in this region of Spain from Equatorial Guinea is clearly lower than that from the rest of sub-Saharan Africa (697 compared to 11,357 in the census on January 1st, 2017) [17]. This evidence has been objectified in the same way in other studies conducted in Spain, with percentages of cases imported from Equatorial Guinea up to 57.1% [8] and 86.26% [7]. In other countries, there has also been a greater number of cases of imported malaria from the corresponding ex-colonies. For example, in France (Marseille) Parola et al. saw a greater number of cases from the Comoros archipelago [12]; in Italy (Milan) Antinori et al. observed the highest percentage of cases from Eritrea [13]; and in Great Britain, Rees et al. found more cases from Nigeria, Ghana and Sierra Leone [14]. This could be due to a greater facility to obtain the nationality, a better knowledge of the language and the sanitary system, or to probable more stable legal and economic conditions. Access to health system in Spain has undergone changes in recent years. At the present time, after Royal Decree-Law 7/2018 [28], emergency assistance is universal, and the necessary requirement to access to public health system is the municipal register (independent of the legal situation). This is controlled by the autonomous communities, which require different minimum registration period to get the health card (most, like Aragon, require 3 months). This means that VFRs have free access to public health system, and newly arrived immigrants only have access to the emergency department until they reach 3 months of registration (for vulnerable groups, there are other specifications in the legislation).

It has been observed a moderate association between Equatorial Guinea and being woman, as well as coming from the rest of sub-Saharan Africa and being man. In the population census of Aragon of January 1st 2017, shows that 63.41% of foreigners from Equatorial Guinea living here are women, compared to 30.04% of women in the group of foreigners from the rest of sub-Saharan Africa [17]. According to United Nations Organization data, female emigration in Equatorial Guinea in 2017 was higher than the male emigration (52.35% versus 47.64%), with Spain being the second country of destination after Gabon. This is the case not only in Equatorial Guinea, as in other African countries such as Democratic Republic of Congo, Côte d’Ivoire, Cameroon, Kenya, Liberia, Namibia or Sierra Leone, female emigration in 2017 was also higher than male migration [29]. Throughout history, migratory movements have been changing. The presence of women in the migration process is increasing [30], and although their social visibility continues to be scarce in relation to the social visibility of immigrant men, research on the feminization of migration is increasing. Women now migrate independently, as the main income generators, instead of migrating accompanying their husbands passively as in the beginning. According to studies, migrant women from sub-Saharan Africa are mostly young and single, migrating in search of work usually from the service sector, such as domestic work and child or elderly care [31]. Despite the fact that female emigration from different African countries is higher than male migration, in Aragon the figures for foreign residents from the rest of sub-Saharan Africa are clearly masculine, unlike Equatorial Guinean figures. The reason for this difference is not known.

This gender distribution between Equatorial Guinea and the rest of sub-Saharan Africa may be the reason for the association between proceeding from Equatorial Guinea and residing in the main province (mostly urban area), as well as between proceeding from sub-Saharan Africa and residing in rural area with important agricultural activity (mainly male activity at present).

The main limitations of this study was the loss of information of the variables reason for travel, fever and chemoprophylaxis in the cases reported in the main province from 1996 to 1999 and in the other provinces from 1996 to 2017. In addition, the epidemiological survey of malaria in Aragon is manual, without mandatory completion of all the variables, in many cases losing data on some of the variables of interest (sometimes probably because of the language barrier).

## Conclusions

The epidemiological profile of malaria cases in Aragon, Spain, has been defined like VFR between 15 and 39 years old, coming from sub-Saharan Africa, particularly from Equatorial Guinea, infected by *P. falciparum*. Almost half of the cases reported in the epidemiological 4-week period 9 to 12.

As in other countries, a greater number of imported cases of malaria from the corresponding ex-colony have been detected, in this case, Equatorial Guinea. It is necessary to analyse more deeply what are the reasons that favor this situation, as well as analyse what factors can influence the fact that cases of other countries that are more populationally represented than the ex-colony are not detected correctly. The cases of malaria from the Spanish ex-colony of Equatorial Guinea, with respect to the cases from the rest of sub-Saharan Africa, have association with the female sex, be under 5 years old, be immigrants and reside in the urban area.

It is necessary to educate immigrants living in developed countries, emphasizing on those who are originally from the ex-colonies of destination country, about the importance of chemoprophylaxis when travelling to visit friends and relatives. Many of the immigrants residing in Spain and, specifically, in Aragon, are usually part of the immigrant association of their country of origin. There are immigrant associations from many countries, including Equatorial Guinea. It would be interesting to start by establishing connections with the association of Equatorial Guineans living in Aragon, to raise awareness of the risk they present when they travel to their country of origin. After that, approach the other immigrant associations from countries in the rest of sub-Saharan Africa. In the same way, it is necessary to empower primary care professionals, who have the opportunity to give advice before trips to tropical areas. Any consultation with an immigrant patient, for whatever reason, especially before summer vacations, provides an opportunity to ask about a possible trip and to inform about the necessary prevention measures.

## Data Availability

The datasets used and/or analysed during the current study are available from the corresponding author on reasonable request.

## Abbreviations

VFR: visiting friends and relatives
IR: interquartile range
OR: odds ratio
CI: confidence interval
